# Volumetric computed tomography with carbon nanotube X-ray source array for improved image quality and accuracy

**DOI:** 10.1038/s44172-023-00123-x

**Published:** 2023-10-07

**Authors:** Shuang Xu, Yuanming Hu, Boyuan Li, Christina R. Inscoe, Donald A. Tyndall, Yueh Z. Lee, Jianping Lu, Otto Zhou

**Affiliations:** 1 Department of Applied Physical Sciences, University of North Carolina at Chapel Hill, Chapel Hill, NC 27599 USA; 2 Department of Physics and Astronomy, University of North Carolina at Chapel Hill, Chapel Hill, NC 27599 USA; 3 Department of Diagnostic Sciences, Adams School of Dentistry, University of North Carolina at Chapel Hill, Chapel Hill, NC 27599 USA; 4 Department of Radiology, University of North Carolina at Chapel Hill, Chapel Hill, NC 27599 USA

**Keywords:** Tomography, Biomedical engineering

## Abstract

Cone beam computed tomography (CBCT) is widely used in medical and dental imaging. Compared to a multidetector CT, it provides volumetric images with high isotropic resolution at a reduced radiation dose, cost and footprint without the need for patient translation. The current CBCT has several intrinsic limitations including reduced soft tissue contrast, inaccurate quantification of X-ray attenuation, image distortions and artefacts, which have limited its clinical applications primarily to imaging hard tissues and made quantitative analysis challenging. Here we report a multisource CBCT (ms-CBCT) which overcomes the shortcomings of the conventional CBCT by using multiple narrowly collimated and rapidly scanning X-ray beams from a carbon nanotube field emission source array. Phantom imaging studies show that, the ms-CBCT increases the accuracy of the Hounsfield unit values by 60%, eliminates the cone beam artefacts, extends the axial coverage, and improves the soft tissue contrast-to-noise ratio by 30–50%, compared to the CBCT configuration.

## all

Xu and colleagues report cone beam computed tomography using a carbon nanotube X-ray source array. The approach addresses the limitations associated with conventional cone beam computed tomography using a single X-ray source, including image distortions, artefacts and low soft tissue contrast.

## Introduction

Cone beam computed tomography (CBCT) has widespread applications in dentistry for clinical tasks including implant and orthodontic treatment planning and evaluation of endodontic conditions of the jaws and teeth^1,2^; and in medical imaging including image-guided radiation therapy, intraoperative surgical guidance, and ENT, extremity and breast imaging^3–6^. Compared to the multidetector CT (MDCT), the current standard for diagnostic imaging^7^, CBCT provides volumetric images with a high isotropic resolution at a relatively low cost, low radiation dose and small footprint, and eliminates the need for patient translation. The large imaging volume of CBCT, however, produces strong scattered radiation that reduces the soft tissue contrast and leads to large errors in quantification of X-ray attenuation^8–10^. The large X-ray cone angle also results in image distortion and artefacts due to insufficient sampling and data truncation which reduce the effective axial coverage. These inherent limitations have restricted the clinical applications of CBCT primarily to imaging hard tissues, and hindered quantitative analyses that are now routinely performed by MDCT, such as accurate determination of the bone mineral density, tissue differentiation, and dose calculation in radiation therapy^11–14^.

To improve the performance of CBCT, various approaches have been proposed and investigated extensively^15–19^. While progresses have been made, the abovementioned issues still remain. Several volumetric CT architectures were designed to address the root cause of these limitations, the large imaging volume, by utilizing multiple X-ray sources. The inverse geometry CT^20,21^ with a narrowly collimated scanning beam and a small area detector and the tetrahedron-beam CT^22^ with orthogonally placed linear source array and detector is effective in rejecting scatter, but still have the off-axis geometry and the X-ray photon fluxes required are difficult to achieve with the current source technologies. An extremity CBCT with three axially placed X-ray tubes each covering the entire field of view (FOV) showed reduced cone beam artefact compared to a regular CBCT^23^. Numerical and experimental simulations demonstrated that the performance of the CBCT could be improved if the single wide cone angle X-ray tube was replaced with multiple collimated sources/beams positioned along the axial axis of the scanner^24,25^. In an experimental simulation study using a single collimated X-ray tube, we showed the multisource CBCT (ms-CBCT) design reduced the scatter-primary-ratio (SPR), increased the uniformity and accuracy of the CT Hounsfield Unit (HU) values, and improved soft tissue contrast for maxillofacial imaging. The ms-CBCT design, however, puts high demands on the X-ray generator, detector, and reconstruction algorithm. An operational ms-CBCT scanner has not been realized till now.

Here, we report a prototype ms-CBCT scanner designed for 3D maxillofacial imaging. The scanner replaces the single wide cone-angle X-ray tube used in the conventional CBCT with a carbon nanotube (CNT) based field emission X-ray source array^26,27^ which generates multiple narrowly collimated and rapidly scanning X-ray beams from multiple closely packed focal spots (“sources”). A residual scatter reduction algorithm enabled by the ms-CBCT data acquisition scheme is implemented to further enhance the image quality. The prototype ms-CBCT was evaluated and compared to the same scanner operating in the conventional CBCT configuration (referred to as “N1 ms-CBCT”) using only one wide cone angle X-ray beam under otherwise the same imaging conditions, the clinical CBCT Accuitomo 170 (Morita, Japan, referred to as “CBCT M”) and the clinical CBCT CS9300 (Carestream Dental, US, referred to as “CBCT C”) at the same kVp and similar imaging dose.

## Results

### Prototype multisource CBCT (ms-CBCT) scanner

**Fig. 1:**
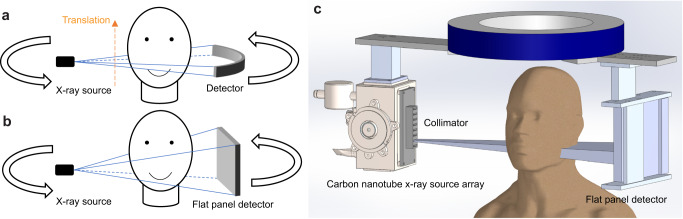
Schematics of MDCT, CBCT and ms-CBCT. **a** A schematic of MDCT. It images only a small section of the object in the axial direction per rotation. The patient needs to be translated for volumetric imaging, and the orange dashed arrow marks the translation direction. **b** With a wider X-ray beam cone angle, the region of interest (ROI) is imaged in one gantry rotation in CBCT. **c** The ms-CBCT replaces the single X-ray tube with an X-ray source array generating multiple narrowly collimated and rapidly scanning X-ray beams, each illuminating a section of the object and collectively covering the entire ROI. (MDCT multidetector computed tomography, CBCT cone beam computed tomography, ms-CBCT multisource cone beam computed tomography).

The prototype scanner consists of a CNT X-ray source array with multiple focal spots (“sources”) aligned along the axial direction of the system and a conventional flat panel detector (FPD), with a system geometry similar to that of a typical dental CBCT, as illustrated in Fig. 1. The radiation from each source is confined to a narrow cone angle by an external multisource collimator to expose only a small section of the object in the axial direction and the full detector width. The FPD was shifted laterally by 70 mm to provide an FOV of 187 mm × 100 mm at the rotation center to cover the entire maxillofacial region of a patient without data truncation.

**Fig. 2:**
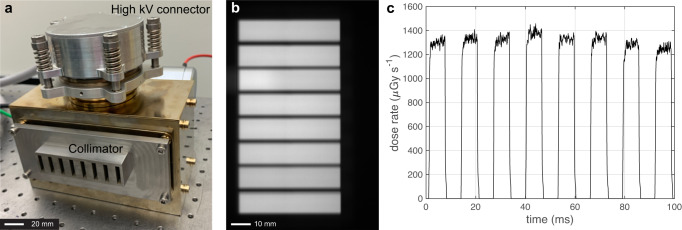
Photograph and characterizations of the carbon nanotube (CNT) field emission X-ray source array. **a** The photograph of the CNT field emission X-ray source array with the external multisource collimator. **b** The radiation fields formed by the eight collimated beams were recorded at 200 mm away from the focal line using a flat panel detector. **c** The X-ray dose rate profiles were measured at the detector surface using a dose meter (Raysafe X2, Unfors Raysafe AB, Sweden), with the source operating at 90 kVp, 15 mA anode current and 6.5 ms exposure per source with 0.3 mm Cu filtration.

The CNT X-ray source array consists of eight gated CNT field emission cathodes and eight corresponding focal spots on an extended tungsten (W) anode with an inter-source spacing of 12 mm, as shown in Fig. 2a. The X-ray exposure and exposure sequence are programmed using an electronic control system (ECS) which automatically adjusts the voltages applied between the individual gate and cathode to achieve and maintain the output. For volumetric CT imaging, the collimated sources are electronically and sequentially scanned across the object with each source activated ${N}_{{{{{{\rm{view}}}}}}}$ times (${N}_{{{{{{\rm{view}}}}}}}$ = 300–500) as the gantry with the array and the FPD rotates by 360°. Each exposure is recorded by one detector frame to collect a total of 8 × *N*_view_ number of projection images.

### X-ray beam profile and dose rate

Figure 2b shows the radiation fields formed by the individual X-ray beams of the CNT X-ray source array after collimation at 200 mm away from the focal line. Each beam was confined to a rectangular field with a measured cone beam angle of $2.4^\circ$, which is comparable to that used in a 64-slice MDCT but smaller than the ~$10^\circ$ cone angle needed to cover the same FOV when the same scanner is operated in the conventional CBCT geometry using only one X-ray source (the “N1 ms-CBCT” configuration). The dose profile measured at the detector surface 615 mm away from the source focal line is shown in Fig. 2c. The average dose rate of the eight sources was 1299 μGy s^−1^ at the exposure condition used in this study. The X-ray pulses show faster rising and damping times (less than 0.5 ms) than those from a typical clinical CBCT with a thermionic X-ray generator. The fast response time minimizes the error in dose calculation and exposure to patients. The dose and dose rate remained constant over the entire ms-CBCT scan of hundreds of exposures per source.

### Quality of the reconstructed phantom images

The image quality and accuracy of the ms-CBCT scanner were investigated by imaging a Contrast phantom, a Defrise phantom, and a RANDO phantom. For comparison, the same phantoms were also imaged using the ms-CBCT scanner operating in the conventional CBCT configuration by using only one source (N1 ms-CBCT) using otherwise the same exposure parameters, the state-of-the-art clinical CBCT M (Accuitomo 170, Morita, Japan) and CBCT C (CS9300 Carestream Dental, US) under similar conditions.

**Fig. 3:**
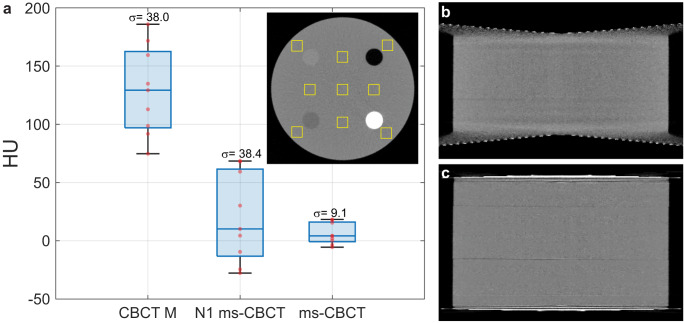
Hounsfield unit (HU) uniformity and sagittal images of the Contrast phantom scanned by the clinical CBCT M, N1 ms-CBCT and ms-CBCT. **a** The boxplot shows the statistical distribution of the SolidWater mean HU values of 9 regions of interest (ROI) marked by yellow solid boxes in the central axial images from the different system configurations. The $\sigma$ is the standard deviation of the HU mean values of 9 ROIs. **b**, **c** The sagittal views of the Contrast phantom measured using the N1 ms-CBCT and ms-CBCT under otherwise the same conditions, respectively. The image distortion caused by data truncation was essentially removed in the ms-CBCT. (Image window: [−1000 HU, 1000 HU], CBCT cone beam computed tomography, ms-CBCT multisource cone beam computed tomography).

The spatial nonuniformity of the SolidWater CT HU values was quantified by measuring the standard deviations of the mean HU values of the nine ROIs placed on the central axial image of the Contrast phantom. As shown in Fig. 3a, the nonuniformity decreased from 38.0 HU for the clinical CBCT M and 38.4 HU for the N1 ms-CBCT to 9.1 HU for the ms-CBCT, representing a 76% decrease in the nonuniformity. The mean HU value for the water equivalent SolidWater measured from the ms-CBCT is 5.8 HU, close to the nominal value of 0. In contrast, the clinical CBCT M and the N1 CBCT show large errors for the same material, by 129 HU and 20 HU, respectively.

Figure 3b, c shows the sagittal images of the Contrast phantom from the N1 ms-CBCT and ms-CBCT, respectively. In the N1 ms-CBCT configuration (conventional CBCT), the top and bottom sections of the image are distorted, which is caused by data truncation due to insufficient detector coverage in the axial direction. In contrast, with the same FPD, this distortion is essentially removed in the ms-CBCT, as shown in Fig. 3c. By reducing the X-ray beam cone angle and therefore the truncation error, the ms-CBCT expands the effective axial FOV from 70 mm in the N1 configuration to 95 mm under otherwise the same geometry.

**Fig. 4:**
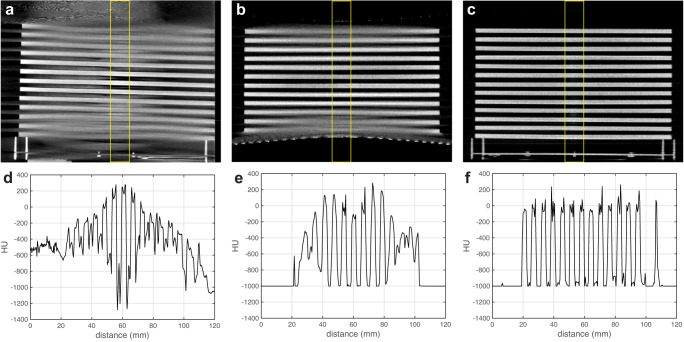
Defrise phantom sagittal images and corresponding line profiles scanned by CBCT M, N1 ms-CBCT, and ms-CBCT. **a**–**c** Sagittal slices of the reconstructed CT images of a homemade Defrise phantom imaged using the clinical CBCT M, N1 ms-CBCT and ms-CBCT. **d**–**f** Corresponding Hounsfield unit (HU) values line profiles measured within the regions of interest marked by yellow solid boxes along the vertical direction. Severe cone beam artefacts were observed in the image from the clinical CBCT scanner, which were essentially eliminated in the image from the ms-CBCT. (Image window: [−1000 HU, 300 HU], CBCT cone beam computed tomography, ms-CBCT multisource cone beam computed tomography).

Figure 4 presents sagittal images and corresponding line profiles of the Defrise phantom scanned using the clinical CBCT M, N1 ms-CBCT and ms-CBCT, respectively. Severe cone beam artefacts in the form of image distortion and blur were observed in the images obtained using the CBCT configuration, due to incomplete sampling in the off-center axial planes^23^. These image blurs and distortions were eliminated in the reconstructed image from the ms-CBCT because of the reduced X-ray beam cone angle. The top surface of the phantom was placed at the upper edge of the effective FOV for the N1 ms-CBCT. Because of the reduced axial coverage from data truncation, the holder plate and the first disc of the Defrise phantom were outside of the FOV in N1 ms-CBCT but were clearly visualized by the ms-CBCT.

**Fig. 5:**
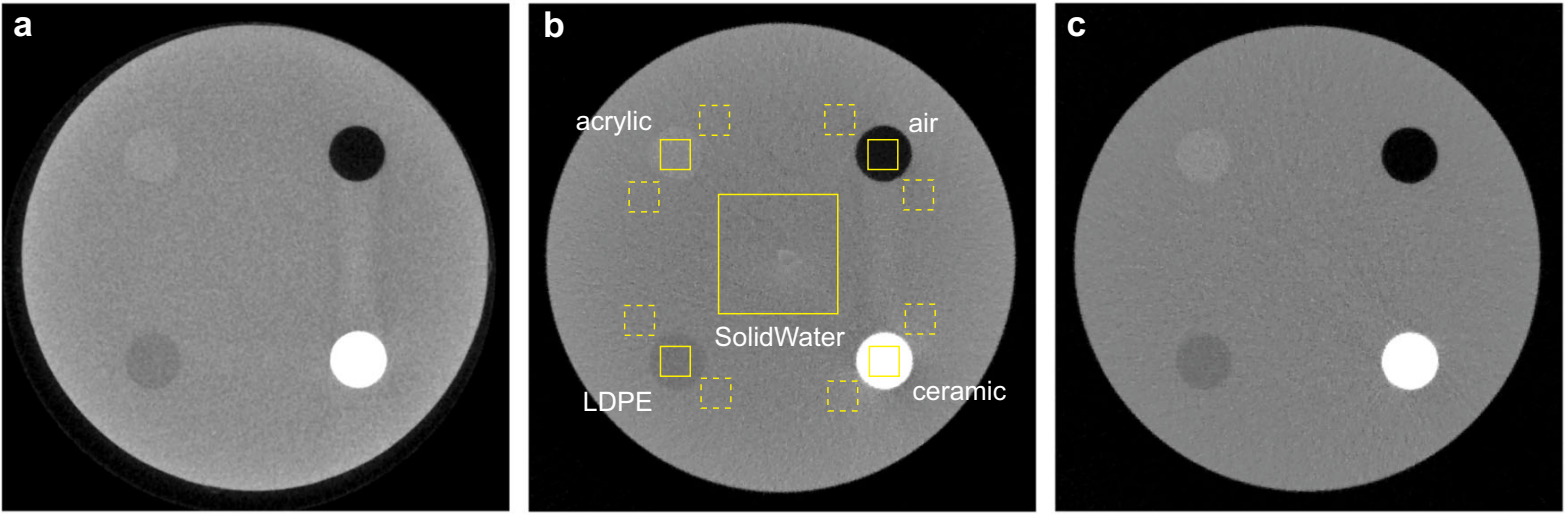
Contrast phantom axial images scanned by the CBCT M, N1 ms-CBCT and ms-CBCT. **a**–**c** The axial images of the Contrast phantom were scanned using the clinical CBCT M, N1 ms-CBCT and ms-CBCT, respectively. The yellow solid boxes were selected to measure the mean Hounsfield unit (HU) values and standard deviations of different materials for the HU accuracy evaluation. The two closest yellow dashed boxes around each insert were selected to measure the mean HU value and standard deviation of their corresponding SolidWater background for contrast and contrast-to-noise ratio calculations. (Image window: [−1000 HU, 1000 HU], CBCT cone beam computed tomography, ms-CBCT multisource cone beam computed tomography, LDPE low-density polyethylene).

Figure 5 shows the central axial images of the Contrast phantom imaged using the clinical CBCT M (Fig. 5a), N1 ms-CBCT (Fig. 5b) and ms-CBCT (Fig. 5c). Visually the image from the ms-CBCT contains fewer artefacts and has a more homogeneous SolidWater background and enhanced contrast compared to the CBCT images. The images from the CBCT M and N1 ms-CBCT appear darker in the center than the periphery, which is the cupping artefact caused by the inhomogeneous distribution of scatter within the scanned volume. Furthermore, the streak and shading artefacts observed around the high-attenuation ceramic resulted from a combination of photon starvation and scatter-induced distortions. As anticipated, the ms-CBCT image exhibited mitigation of these artefacts because of the substantial scatter reduction.

Quantitative analysis was performed to compare the contrast and contrast-to-noise ratio (CNR) using the data from the N1 ms-CBCT and ms-CBCT as the two used the same exposure parameters and reconstruction algorithm except the N1 ms-CBCT has the conventional CBCT configuration. As shown in Fig. 5b, four 10 mm × 10 mm solid squares were selected to measure the mean HU value of four different inserts, and two 10 mm × 10 mm dashed squares near each insert were used to measure the mean HU value and standard deviation of the SolidWater background associated with each insert respectively. This method of selecting ROIs could effectively decouple the impact of the cupping effect by ensuring the measurements of the inserts and their corresponding SolidWater background were taken at the same radius. The contrast was calculated as the absolute HU difference between the insert and its background, and the CNR was calculated as the contrast of the insert divided by the standard deviation of its SolidWater background. The results of all four inserts were summarized in Table 1. Compared to N1 ms-CBCT, ms-CBCT increased the contrast by ~20%, and enhanced the CNR by about 30–50%.

**Table 1:** Contrasts and contrast-to-noise ratios (CNR) of different inserts measured from the Contrast phantom scanned by the N1 ms-CBCT and ms-CBCT under the same exposure conditions.

	N1 ms-CBCT		ms-CBCT		Improvement	
Inserts	contrast	CNR	contrast	CNR	contrast	CNR
Arcylic	72.1 HU	1.52	89.9 HU	2.34	24.6%	53.4%
LDPE	100.1 HU	2.08	126.5 HU	2.88	26.4%	38.1%
Air	825.8 HU	17.70	967.7 HU	24.10	17.2%	36.2%
Ceramic	1542.5 HU	28.35	2094.3 HU	44.25	35.8%	56.1%

*ms-CBCT* multisource cone beam computed tomography.

### Accuracy of the CT HU value

The mean HU values and the standard deviations of various inserts and the SolidWater of the Contrast phantom were measured by averaging over the solid ROIs shown in Fig. 5b. The results are listed in Table 2. The results from the same Contrast phantom measured using a clinical MDCT (SOMATOM Force, Siemens, Germany) were included as the reference. As shown, the HU values derived from the N1 ms-CBCT and the clinical CBCT M both deviate from the MDCT reference, consistent with previous studies of CBCT^28–30^. The deviations are reduced in results from the ms-CBCT. The root-mean-square error (RMSE) of the HU values for the five materials is reduced from 385 HU in N1 ms-CBCT and 316 HU in the clinical CBCT M to 145 HU in the ms-CBCT.

**Table 2:** Hounsfield unit (HU) values of the Contrast phantom measured from CBCT M, N1 ms-CBCT, ms-CBCT, and clinical MDCT.

	CBCT M	N1 ms-CBCT	ms-CBCT	MDCT	Nominal value
	HU	HU	HU	HU	HU
Acrylic	$160.2\pm 42.3$	$66.7\pm 44.0$	$87.5\pm 41.2$	$123.8\pm 27.1$	$120$
Air	$-867.6\pm 44.1$	$-849.7\pm 30.2$	$-973.6\pm 19.2$	$-1023.3\pm 0.9$	$-1000$
Macor	$1744.2\pm 70.5$	$1583.3\pm 82.9$	$2105.2\pm 79.8$	$2423.3\pm 42.1$	
LDPE	$-25.8\pm 44.5$	$-103.3\pm 41.5$	$-126.3\pm 34.1$	$-119.6\pm 22.0$	$-95$
Water	$87.7\pm 44.0$	$-27.6\pm 55.5$	$-11.5\pm 47.7$	$15.4\pm 24.7$	$0$
RMSE	316	385	145		

Note: (1) Macor is a machinable glass ceramic composed of ~55% fluorophlogopite mica and 45% borosilicate glass. (2) The nominal values are provided by the standard CT ACR 464 phantom which is suggested for 120–130 kVp^39,43^ rather than the 90 kVp used in this study.

*CBCT* cone beam computed tomography, *ms-CBCT* multisource cone beam computed tomography, *MDCT* multidetector computed tomography, *LDPE* low-density polyethylene, *RMSE* root-mean-square error.

### Image quality and accuracy improvement for maxillofacial imaging

**Fig. 6:**
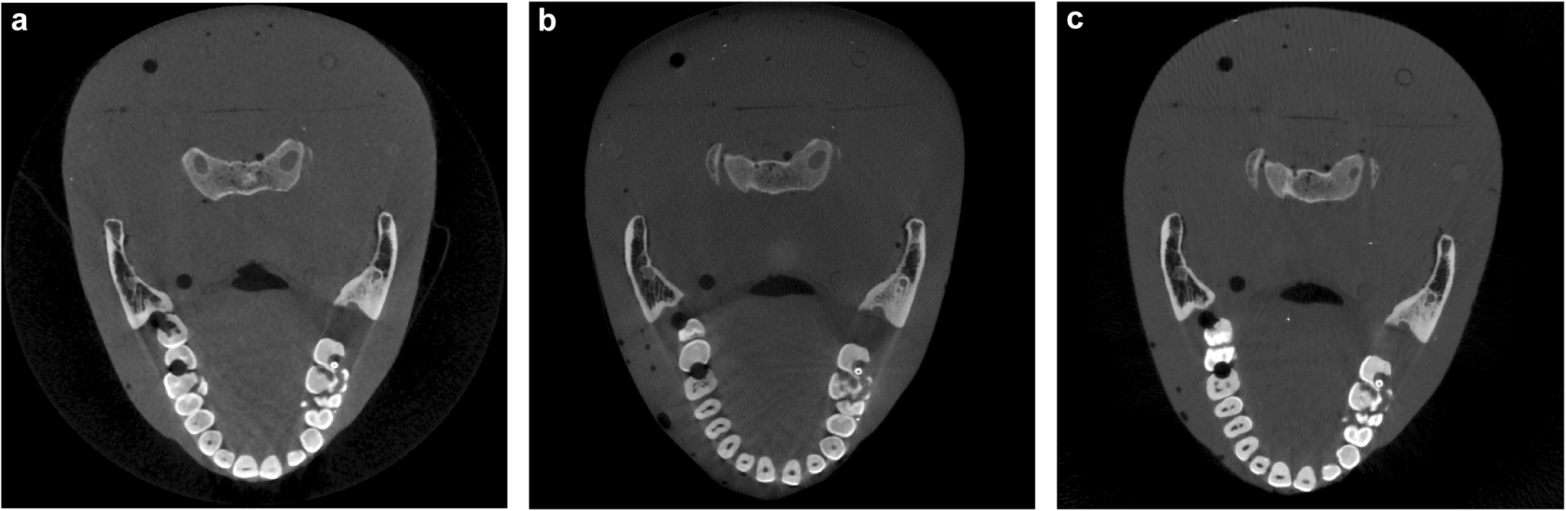
RANDO phantom axial images scanned by the CBCT M, CBCT C and ms-CBCT. **a**–**c** The axial images of the Contrast phantom scanned using the clinical CBCT M, clinical CBCT C and ms-CBCT, respectively. (Image window: [−1000 HU, 3000 HU]), CBCT cone beam computed tomography, ms-CBCT multisource cone beam computed tomography).

Figure 6 shows the axial images of the RANDO phantom scanned by the CBCT M, CBCT C, and ms-CBCT. The severe shading and streak artefacts around the bones and teeth shown in clinical CBCTs were reduced in the ms-CBCT due to the substantial scatter reduction.

**Fig. 7:**
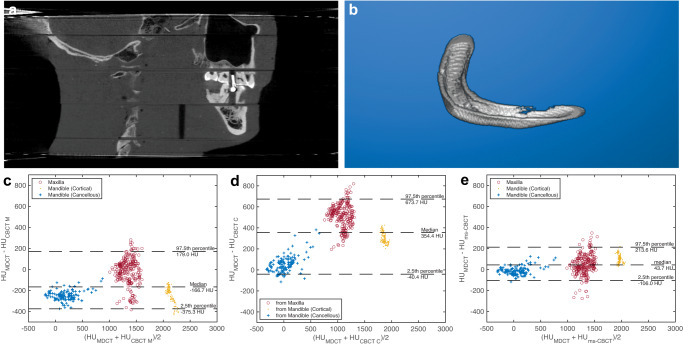
RANDO phantom sagittal image scanned by the ms-CBCT, segmented cortical bone of the mandible and Bland-Altman (BA) plots. **a** The sagittal view of the RANDO phantom scanned by the ms-CBCT. **b** Segmented cortical bones of the mandible. **c**–**e** BA plots of the difference between averaged Hounsfield unit (HU) values of the registered regions of interest from the MDCT and the clinical CBCT M, the clinical CBCT C and ms-CBCT, respectively. The median difference, 2.5th and 97.5th percentile were used for non-normally distributed data and calculated based on all 382 data points. Note: The discontinuities shown in Fig. 7a were caused by air gaps and misalignments between different slabs of the RANDO phantom (Image window: [−1000 HU, 3000 HU], ms-CBCT: multisource cone beam computed tomography, MDCT: multidetector computed tomography, CBCT: cone beam computed tomography).

To compare the HU values measured from the mandible and the maxilla bones of the RANDO phantom, the reconstructed volumes from all used scanners were first registered under the rigid body condition using an open-source software 3D Slicer^31^ to ensure the same ROIs were selected. The cortical and cancellous bones of the mandible and a section of the maxilla were then segmented using the threshold method. Figure 7a is the sagittal image of the RANDO phantom scanned by the ms-CBCT. The discontinuities in the image were caused by the air gaps and misalignments between different slabs of the phantom. The registration and segmentation were conducted using one slab of the head phantom. Figure 7b shows the segmented cortical bones of the mandible. Each sagittal slice (0.3 mm in thickness) of the segmented image was used as one ROI, resulting in a total of 131 ROIs in the mandible and 251 ROIs in the maxilla. The averaged HU numbers for each ROI were obtained and statistical analysis was performed. The results for the three different types of bones, which have a non-normal data distribution, are presented in a Bland-Altman (BA) plot of the difference versus the mean HU value in Fig. 7c–e.

The median difference in the HU values between the MDCT and the clinical CBCT M was found to be −166.7 HU, and the 2.5th and 97.5th percentile lines were −375.3 HU and 179.0 HU. The results of the clinical CBCT C showed a median difference 354.4 HU, and the 2.5th and 97.5th percentile lines were −40.4 HU and 673.7 HU. The agreement with the MDCT was improved for the ms-CBCT. The median difference was found to be 43.7 HU, and the 2.5th and 97.5th percentile lines were −106.0 and 213.6 HU.

## Discussion

The results presented here demonstrate the ms-CBCT design improves the image quality and accuracy compared to the conventional CBCT. The imaging dose used by the ms-CBCT is in the range of that by clinical CBCTs for the same object, which varies with the manufacturer, model and imaging protocol^32,33^. At 11.8 dGy cm^2^ DAP, the dose of the ms-CBCT with the detector offset geometry was calculated to be 6.3 mGy at the rotation center, similar to the 9.0 mGy dose for the clinical CBCT C based on the 19.8 dGy cm^2^ DAP.

One limitation of the current prototype is the increased scanning time, which is determined by:

1 $${T}_{{scan}}=\left({\Delta t}_{{{{{{\rm{readout}}}}}}}+{\Delta t}_{{{{{{\rm{exposure}}}}}}}\right)\times {N}_{{{{{{\rm{view}}}}}}}\times {N}_{{{{{{\rm{source}}}}}}}$$

Where the ${\Delta t}_{{{{{{\rm{readout}}}}}}}$ is the detector readout time and ${\triangle t}_{{{{{{\rm{exposure}}}}}}}$ is the exposure time per X-ray pulse. For the imaging protocol used in this study and the readout speed of the present FPD, ${T}_{{{{{{\rm{scan}}}}}}}$ is 52 s. The longer than desired imaging time is partially caused by the inefficiency of photon utilization in the offset geometry using a narrow FPD. One way to mitigate this issue is to increase the detector width to cover the entire FOV without the offset while keeping other parameters the same. This will reduce the scanning time to 26 s without compromising the performance, which is in the time range of the current clinical CBCT^32,33^. Further reduction of the scanning time can be achieved by using an FPD with a faster readout speed, optimization of the number of projection views and the imaging dose.

In conclusion, the results of this study demonstrate that the ms-CBCT design with a spatially distributed X-ray source array improves the image quality and the diagnostic accuracy of volumetric CT. The scanner is made possible by the CNT field emission X-ray source array technology which generates multiple X-ray beams that can be readily programmed and rapidly scanned with the resolution, consistency, stability and dose rate required for maxillofacial imaging. Phantom imaging studies show it reduces the spatial nonuniformity and RMSE of the CT HU values by respectively 75% and 60%, essentially eliminates the cone beam artefacts, increases the effective axial coverage, and improves CNR of different materials by 30% ~ 50%. It increases the accuracy and reliability of measuring the mandible and maxilla bones compared to the state-of-the-art clinical CBCT. These improvements have the potential to bring the diagnostic accuracy of CBCT to a level comparable or close to that of an MDCT, without changing its important attributes including volumetric imaging at low radiation, low cost, and small footprint. It can potentially expand the clinical applications of CBCT from primarily imaging hard tissue to clinical tasks commonly performed by the more costly and bulky MDCT, including quantitative analysis.

## Methods

### CNT X-ray source array

The CNT X-ray source array (NuRay Technology Co., Ltd., Changzhou, China) was specifically designed for this scanner and consists of eight gated CNT field emission cathodes and eight corresponding focal spots on an extended W anode with an inter-source spacing of 12 mm. The entire assembly is enclosed in a stainless housing with a 1.7 mm thick Al window which also serves as the inherent filtration. The averaged focal spot size, measured by the standard pin-hole method, is 0.88 mm (width) × 1.10 mm (height) at the 15% maximum intensity with a standard deviation of 0.05 mm (width) × 0.04 mm (height) for the eight spots^34^. The spot size is comparable to that commonly used in a clinical dental CBCT^32^.

### Flat panel detector (FPD)

The FPD (Xineos-1511 from Teledyne DALSA in Waterloo, CA) is a CMOS flat panel detector with a CsI scintillator designed for dental CBCT. It has an active area of 147.3 mm (width) × 113.7 mm (height) and a pixel pitch of 99 μm. The FPD was operated in the 2 × 2 binning mode with a readout time ${t}_{{{{{{\rm{readout}}}}}}}$ of 11.6 ms per frame.

### Adjacent scatter ratio subtraction

The projection images are processed using an adjacent scatter ratio subtraction (ASRS) method specifically for the ms-CBCT geometry. It utilizes the scatter ${I}_{{{{{{\rm{s}}}}}}}^{{\prime} }$ recorded on the adjacent director rows not exposed by the primary beam ${I}_{{{{{{\rm{p}}}}}}}$ to estimate and remove the residual scatter ${I}_{{{{{{\rm{s}}}}}}}$ in each projection image. To minimize contamination from the primary beam, 50 detector rows centered 50 mm away from the center of the illuminated area are averaged to obtain the ${I}_{{{{{{\rm{s}}}}}}}^{{\prime} }$ as a function of the detector column number. The adjacent scatter ratio $r$, defined as ${I}_{{{{{{\rm{s}}}}}}}^{{\prime} }/({I}_{{{{{{\rm{p}}}}}}}+{I}_{{{{{{\rm{s}}}}}}})$, is calculated for each column. A spline function is used to fit the raw data to obtain the smoothed ratio ${r}^{{\prime} }$. The primary photons ${I}_{{{{{{\rm{p}}}}}}}$ in the exposed region are estimated by applying $({I}_{{{{{{\rm{p}}}}}}}+{I}_{{{{{{\rm{s}}}}}}})(1-{r}^{{\prime} })$. The same ${r}^{{\prime} }$ function is applied to for all detector rows in the directly illuminated area to subtract the residual scatter. This approach was found to be effective in further reducing the scatter radiation in our feasibility study^35^.

### Image reconstruction and calibration

For N1 ms-CBCT and ms-CBCT, the processed projections from all sources are used for volumetric CT reconstruction using the 3D Simultaneous Iterative Reconstruction Technique algorithm based on the ASTRA Toolbox^36^. The reconstruction is performed using a single system matrix, including the source/detector rays from all sources. The total variation (TV) algorithm is incorporated for image noise reduction based on the Tigre Toolbox^37,38^. The CT numbers of all reconstructions are calibrated according to Gammex CT ACR 464 phantom testing instruction^39^. The images from the clinical CBCT M and MDCT were reconstructed by the manufacturer-specific software packages.

### Phantoms

The Defrise phantom consists of a stack of acrylic discs of 160 mm diameter × 4 mm thickness with a 4 mm air gap. The homemade cylindrical Contrast phantom consists of a 16 cm diameter water equivalent plastic material (SolidWater, Sun Nuclear Co, Melbourne FL) with four wells filled with acrylic, low-density polyethylene, air, and Macor (machinable ceramic) sandwiched between uniform SolidWater discs of the same diameter. The RANDO phantom is an anthropomorphic adult skull and tissue equivalent head phantom (RANDO, Nuclear Associates, NY).

### DAP calculation

The dose area product (DAP) of the ms-CBCT was calculated using the experimentally measured dose rate at the detector surface and the detector area illuminated by the primary beam per exposure, which is the detector width (147.3 mm) × full width half maximum of the primary beam in the axial direction (32.5 mm), following Eq. (2).

2 $${DAP}=D\times \Delta {t}_{{{{{{\rm{exposure}}}}}}}\times A\times {N}_{{{{{{\rm{view}}}}}}}\times {N}_{{{{{{\rm{source}}}}}}}$$

where $D$ is the dose rate, $\triangle {t}_{{{{{{\rm{exposure}}}}}}}$ is the exposure time per X-ray pulse, $A$ is the area of the detector segment illuminated by the primary photons per exposure, ${N}_{{{{{{\rm{view}}}}}}}$ is the number of views and ${N}_{{{{{{\rm{source}}}}}}}$ is the number of sources, which are 360 and 8, respectively in this study.

### Scanner parameters

Four scanners were used in this study, including the clinical CBCT M (Accuitomo 170, Morita, Japan), the clinical CBCT C (CS9300, Carestream Dental, US), the benchtop ms-CBCT and the clinical MDCT (SOMATOM Force, Siemens, Germany). Their geometry and exposure parameters are summarized in Table 3 below. The N1 ms-CBCT is the ms-CBCT scanner operating in the conventional CBCT configuration, using only one of its sources and the same exposure parameters. The CBCT C malfunctioned during this study, only results from the RANDO phantom are available from this instrument.

**Table 3:** Exposure and geometry parameters of the clinical CBCT M and CBCT C, benchtop ms-CBCT, and clinical MDCT used in this study.

	CBCT M	CBCT C	ms-CBCT	MDCT
Tube voltage	90 kVp	90 kVp	90 kVp	90 kVp
Half-value layer	5.5 mm Al	4.9 mm Al	6.7 mm Al	7.3 mm Al
Tube current	6 mA	5 mA	15 mA	85/72 mA
Exposure	105 mAs	56.5 mAs	35.1 mAs	100/90 mAs
Radiation mode	continuous	pulsed	pulsed	pulsed
Field of view	170 mm × 120 mm	170 mm × 130 mm	187 mm × 100 mm	N.A.
Dose area product	30.7 dGy·cm^2^	19.8 dGy·cm^2^	11.8 dGy·cm^2^	N.A.
Averaged dose	15.0 mGy	9.0 mGy	6.3 mGy	N.A.

Note: (1) The half-value layer thickness of CBCT C and MDCT were simulated with Spektr 3.0^44^. (2) For the MDCT, 85 mA and 100 mAs were used for the Contrast phantom, and 72 mA and 90 mAs were used for the RANDO phantom.

*CBCT* cone beam computed tomography, *ms-CBCT* multisource cone beam computed tomography, *MDCT* multidetector computed tomography.

### Statistics and reproducibility

To measure the HU spatial nonuniformity, we selected nine regions of interest (ROIs) consisting of 20 × 20 pixels each. The mean value for all pixels within each ROI was calculated, and the standard deviation of these nine mean values was then determined and presented in the boxplot Fig. 3a. For measuring the CNR and HU accuracy, we selected 4 ROIs with 20 ×  20 pixels, as well as one larger ROI consisting of 80 × 80 pixels. The mean HU value for each ROI was determined, and the standard deviation of all pixels within corresponding ROIs was calculated and recorded in Table 2. To compare the agreement with the MDCT for mandible and maxilla bone measurements, the MDCT reconstructed volume was used as the reference to create Bland-Altman plots. Prior to analysis, image registration and segmentation were conducted to exclude orientation differences. The sagittal views for the mandible and maxilla consisted of 131 and 251 slices. The mean HU value for each slice of two scanners was used as the horizontal axis. The difference in each slice between two scanners was used as the vertical axis. The data for the three different types of bones do not follow the normal distribution. A variation of the BA plot for non-normal distribution data was used^40–42^. The middle line represents the median difference between two scanners. The lower and upper lines represent the boundary of the 2.5th and 97.5th percentile.

## Data Availability

The data that support the findings of this study are available from the corresponding author upon reasonable request.
